# Evaluation of safety profile of thoracoscopic esophagectomy for T1bN0M0 cancer using data from JCOG0502: a prospective multicenter study

**DOI:** 10.1007/s00464-015-4102-4

**Published:** 2015-02-13

**Authors:** Isao Nozaki, Ken Kato, Hiroyasu Igaki, Yoshinori Ito, Hiroyuki Daiko, Masahiko Yano, Harushi Udagawa, Junki Mizusawa, Hiroshi Katayama, Kenichi Nakamura, Yuko Kitagawa

**Affiliations:** 1 Department of Surgery, Shikoku Cancer Center Hospital, 160 Minami-umemoto, Matsuyama, 791-0280 Japan; 2 Department of Gastrointestinal Medical Oncology, National Cancer Center Hospital, Tokyo, Japan; 3 Department of Esophageal Surgery, National Cancer Center Hospital, Tokyo, Japan; 4 Department of Radiation Oncology, National Cancer Center Hospital, Tokyo, Japan; 5 Department of Esophageal Surgery, National Cancer Center Hospital East, Chiba, Japan; 6 Department of Surgery, Osaka Medical Center for Cancer and Cardiovascular Diseases, Osaka, Japan; 7 Department of Gastroenterological Surgery, Toranomon Hospital, Tokyo, Japan; 8 JCOG Data Center/Operations Office, Center for Research Administration and Support, National Cancer Center, Tokyo, Japan; 9 Department of Surgery, Keio University School of Medicine, Tokyo, Japan

**Keywords:** Esophagectomy, Thoracoscopy, Laparoscopy, Reoperation, Minimally invasive surgery, Esophageal cancer

## Abstract

**Background:**

Thoracoscopic esophagectomy is rapidly and increasingly being used worldwide because it is a less invasive alternative to open esophagectomy. However, few prospective multicenter studies have evaluated its safety profile. This study aimed to evaluate the safety profile of thoracoscopic esophagectomy using perioperative data from the Japan Clinical Oncology Group Study (JCOG0502).

**Methods:**

JCOG0502 is a four-arm prospective study comparing esophagectomy with chemoradiotherapy for esophageal cancer, with randomized and patient preference arms. Patients with clinical stage T1bN0M0 esophageal cancer were enrolled until patient accrual was completed. Open or thoracoscopic esophagectomy was selected at the surgeon’s discretion. Perioperative complications were defined as adverse events of ≥grade 2 as per Common Terminology Criteria for Adverse Events ver. 3.0.

**Results:**

A total of 379 patients were enrolled between December 2006 and February 2013. Of the 210 patients who underwent surgery, 109 patients underwent open esophagectomy, and 101 patients underwent thoracoscopic esophagectomy. Although thoracoscopic esophagectomy decreased the incidence of postoperative atelectasis (open: 22.0 %, thoracoscopy: 10.9 %; *P* = 0.041), reoperation was more frequent in the thoracoscopy group (open: 1.8 %, thoracoscopy: 9.9 %; *P* = 0.016). The incidence of overall complications did not differ between the two groups (open: 44.0 %, thoracoscopy: 44.6 %; *P* = 1.00). There was one in-hospital death in each group (open: 0.9 %, thoracoscopy: 1.0 %; *P* = 1.00).

**Conclusions:**

Thoracoscopic esophagectomy is a safe procedure with morbidity and mortality comparable with those of open esophagectomy. However, it is associated with a higher frequency of reoperation.

Esophagectomy remains the only potentially curative treatment for thoracic esophageal cancer. It can be performed via either the transthoracic or transhiatal approach. The first transthoracic esophagectomy for cancer was performed through a thoracotomy by Franz Torek in 1913 [1]. Since then, majority of transthoracic esophagectomies have been performed through a thoracotomy until Cuschieri et al. [2] first introduced thoracoscopic esophagectomy in 1992. Several systematic reviews and meta-analyses have shown that thoracoscopic esophagectomy is associated with decreased blood loss and shorter hospital and intensive care unit stays [3–5]. These positive findings have contributed to the rapid increase in the use of thoracoscopic esophagectomy worldwide [6, 7]. Indeed, one-third of all transthoracic esophagectomies performed in Japan during 2011 utilized the thoracoscopic approach [8]. Despite its widespread use in recent years, few prospective multicenter studies have evaluated the safety profile of thoracoscopic esophagectomy [9, 10].

The 5-year survival of patients with stage I thoracic esophageal cancer is 70–80 %, regardless of whether they underwent esophagectomy or definitive chemoradiotherapy [11–13]. Therefore, we conducted a prospective multicenter phase III study: Japan Clinical Oncology Group Study 0502 (JCOG0502), in which we compared these two treatments in this patient population, and a primary analysis of overall survival is planned in 2018. The present study aimed to evaluate the safety profile of thoracoscopic esophagectomy in comparison with open esophagectomy using perioperative data from the JCOG0502 study.

## Materials and methods

### Study design and patient selection

**Fig. 1:**
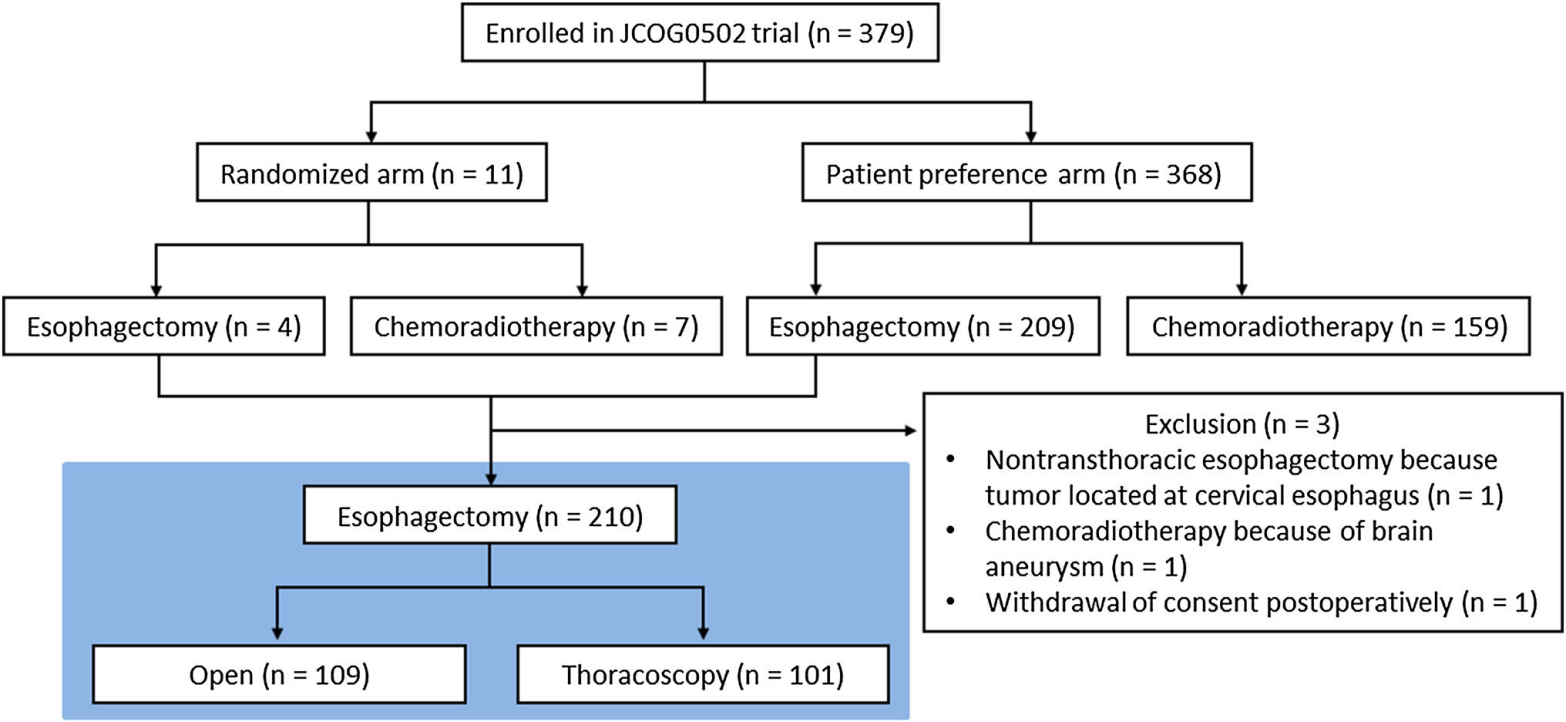
Flow diagram for the Japan Clinical Oncology Group (JCOG) trial 0502, with the present study highlighted in blue

JCOG0502 is a four-arm prospective study comparing esophagectomy with definitive chemoradiotherapy for esophageal cancer, with randomized and patient preference arms [14]. In this study design, if patients accepted randomization because they had no strong preference, they were randomly allocated to one of the two treatments (Fig. 1). However, if patients had a strong preference and therefore refused randomization, they were allocated to the arm with their preferred treatment. Written informed consent was obtained from all enrolled patients. The study protocol was approved by the Clinical Trial Review Committee of the JCOG and by review boards of all the participating institutions. This study was registered with UMIN-CTR (www.umin.ac.jp/ctr/) (registration number: UMIN000000551). Key eligibility criteria for JCOG0502 were that patients should be aged between 20 and 75 years and diagnosed with histologically proven squamous cell carcinoma, adenosquamous cell carcinoma, or basaloid cell carcinoma in the thoracic esophagus of clinical stage IA (T1bN0M0) according to the seventh edition of the UICC TNM staging system [15] and Eastern Cooperative Oncology Group performance status 0–1. Patient accrual for this study was completed. The primary endpoint is overall survival in the randomized arm, which is planned to be analyzed in 2018. Secondary endpoints are overall survival in the patient preference arm, complete response rate after definitive chemoradiotherapy, and adverse events and progression-free survival of all patients.

### Operative methods

After patients were allocated to the surgery arms, subtotal esophagectomy with lymphadenectomy was performed without preoperative chemotherapy and/or radiotherapy. Open or thoracoscopic esophagectomy was selected at the surgeon’s discretion. Open esophagectomy was performed via a right thoracotomy in the lateral decubitus position followed by either the laparotomic or laparoscopic approach. Thoracoscopic esophagectomy was performed through a right thoracoscopy in the lateral decubitus or prone position followed by either the laparotomic or laparoscopic approach. The anastomotic site and technique were selected according to the standard of each participating institution. Patients with upper thoracic disease underwent three-field lymphadenectomy, whereas patients with mid- or lower thoracic disease underwent either two-field or three-field lymphadenectomy at the surgeon’s discretion.

### Definitions

Perioperative adverse events and laboratory abnormalities were graded according to Common Terminology Criteria for Adverse Events ver. 3.0 (CTCAE v3.0) [16]. Perioperative complications were defined as adverse events of ≥grade 2 as per CTCAE v3.0. Postoperative mortality was defined as postoperative death due to any cause within 30 days or death during the same hospital admission. Reoperation was defined as any secondary surgery under general anesthesia during the same hospital admission.

### Statistical methods

Our planned sample size for the randomized arm was 57 patients per arm. The planned sample size for the patient preference arms was at least 156 patients per arm. The sample size for each arm was calculated to demonstrate that the overall survival of the chemoradiotherapy arms was noninferior compared with that of the esophagectomy arms. For evaluating the safety profile of thoracoscopic esophagectomy, perioperative morbidity and mortality were compared with those of open esophagectomy. In addition, the frequency of reoperation and laboratory abnormalities were also compared. To compare data between the two groups, the Wilcoxon rank sum test was used for continuous data and the Fisher’s exact test for categorical data. The level of significance was set at a two-sided *P* value of <0.05. All analyses were performed using SAS software, ver. 9.2 (SAS Institute Inc., Cary, NC) at the JCOG Data Center. The data presented in this article include those up to November 2013.

## Results

### Patient characteristics and operative details

A total of 379 patients with clinical stage IA (T1bN0M0) esophageal cancer were enrolled in the JCOG0502 trial from December 2006 to February 2013 from 37 institutions (Fig. 1). Among the 379 patients, 11 were allocated to the randomized arm, and 368 were allocated to the patient preference arm. Excluding one patient who withdrew consent postoperatively, 210 of 379 patients underwent esophagectomy. Of these 210 patients, 109 underwent open esophagectomy, and 101 underwent thoracoscopic esophagectomy. As shown in Table 1, open esophagectomies were combined with the open abdominal approach in 102 of the 109 (94 %) patients, whereas thoracoscopic esophagectomies were combined with the laparoscopic approach in 58 of the 101 (57 %) patients. Majority of patients underwent gastric pull-up reconstruction (*n* = 206), with the colon being used as a conduit in the remaining patients (*n* = 4). For these reconstructions, the retrosternal route was more often selected in the open group, whereas the posterior mediastinal route was more often selected in the thoracoscopy group. Blood loss was less in the thoracoscopy group, and operating time was shorter in the open group. Despite the difference in blood loss, there was no obvious difference between the groups regarding the frequency of red blood cell transfusion.

**Table 1:** Patient characteristics and operative details

	Open (*n* = 109)		Thoracoscopy (*n* = 101)		*P* ^a^
	*n*	%	*n*	%	
Age (years)					
Median (range)	62 (41–75)		63 (48–75)		0.522^b^
Gender					
Male	93	85.3	82	81.2	0.462
Female	16	14.7	19	18.8	
Body mass index					
Median (range)	22 (13–29)		23 (17–28)		0.934^b^
Tumor location					
Upper thoracic	12	11.0	15	14.9	0.183
Mid-thoracic	65	59.6	67	66.3	
Lower thoracic	32	29.4	19	18.8	
Tumor size					
≤4 cm	76	69.7	70	69.3	1.000
>4 cm	33	30.3	31	30.7	
Lymphadenectomy					
Two-field	41	37.6	40	39.6	0.779
Three-field	68	62.4	61	60.4	
Abdominal approach					
Open	102	93.6	43	42.6	<0.0001
Laparoscopy	7	6.4	58	57.4	
Reconstruction route					
Ante-sternal	6	5.5	0	0	<0.0001
Retrosternal	50	45.9	23	22.8	
Posterior mediastinal	53	48.6	78	77.2	
Blood loss (mL)					
Median (range)	412 (45–1,833)		293 (0–4,225)		<0.001^b^
Operating time (min)					
Median (range)	399 (222–638)		510 (310–871)		<0.0001^b^
Red blood cell transfusion					
Yes	5	4.5	3	3.0	0.723
Lymph nodes harvested					
Median (range)	47 (19–120)		56 (18–120)		0.063^b^

^a^Fisher’s exact test

^b^Wilcoxon rank sum test

### Perioperative morbidity and mortality

Perioperative complications and other outcomes are shown in Table 2. The proportion of intraoperative complications was similarly low in both groups (open: 2.8 %, thoracoscopy: 3.0 %; *P* = 1.00). The four most common postoperative complications were atelectasis, recurrent nerve palsy, pneumonia, and anastomotic leak, with the incidences of 17, 15, 12, and 10 %, respectively. Although the frequency of overall postoperative complications did not differ significantly between the two groups (open: 44.0 %, thoracoscopy: 44.6 %; *P* = 1.00), the incidence of atelectasis was lower in the thoracoscopy group (open: 22.0 %, thoracoscopy: 10.9 %; *P* = 0.041). Although the incidence of pneumonia was also lower in the thoracoscopy group, the difference was insignificant (open: 15.6 %, thoracoscopy: 7.9 %; *P* = 0.093). Patients in the thoracoscopy group were further subdivided on the basis of whether procedures were performed in the prone (*n* = 40) or the lateral decubitus position (*n* = 61). The incidences of atelectasis were 10 and 11 % in the prone and lateral decubitus positions, respectively, whereas those of pneumonia were 10 and 7 %, respectively.

**Table 2:** Perioperative complications and other outcomes

	Open (*n* = 109)		Thoracoscopy (*n* = 101)		*P* ^a^
	*n*	%	*n*	%	
Intraoperative complications	3	2.8	3	3.0	1.000
Postoperative complications (any)	48	44.0	45	44.6	1.000
Pulmonary					
Atelectasis	24	22.0	11	10.9	0.041
Pneumonia	17	15.6	8	7.9	0.093
Recurrent nerve palsy	17	15.6	15	14.9	1.000
Anastomotic leak	15	13.8	7	6.9	0.120
Intravascular catheter infection	4	3.7	2	2.0	0.684
Paralytic ileus	2	1.8	3	3.0	0.673
Intestinal obstruction	0	0	4	4.0	0.052
Other	5	4.6	16	15.8	0.010
Reoperation	2	1.8	10	9.9	0.016
Postoperative mortality	1	0.9	1	1.0	1.000
Postoperative length of stay (days)					
Median (range)	22 (10–162)		24 (9–185)		0.472^b^

^a^Fisher’s exact test

^b^Wilcoxon rank sum test

Ninety-three patients had one or more postoperative complications. Associations between overall complications and baseline characteristics are shown in Table 3; however, no significant risk factor was detected.

**Table 3:** Risk factors for postoperative complication and reoperation

	Total (*n* = 210)	Any complication (*n* = 93)			Reoperation (*n* = 12)		
	*n*	*n*	%	*P* ^a^	*n*	%	*P* ^a^
Age				0.78			0.54
<65	129	56	43.4		6	4.7	
≥65	81	37	45.7		6	7.4	
Gender				1.00			1.00
Male	175	78	44.6		10	5.7	
Female	35	15	42.9		2	5.7	
Body mass index				0.73			0.71
<25	168	73	43.5		9	5.4	
≥25	42	20	47.6		3	7.1	
Tumor location				0.43			0.093
Upper thoracic	27	14	51.9		4	14.8	
Mid-thoracic	132	60	45.5		5	3.8	
Lower thoracic	51	19	37.3		3	5.9	
Tumor size				0.29			0.11
≤4 cm	146	61	41.8		11	7.5	
>4 cm	64	32	50.0		1	1.6	
Lymphadenectomy				0.20			0.54
Two-field	81	31	38.3		6	7.4	
Three-field	129	62	48.1		6	4.7	
Thoracic approach				1.00			0.016
Open	109	48	44.0		2	1.8	
Thoracoscopy	101	45	44.6		10	9.9	
Abdominal approach				0.88			0.20
Open	145	65	44.8		6	4.1	
Laparoscopy	65	28	43.1		6	9.2	

^a^Fisher’s exact test

There was one in-hospital death in each group (open: 0.9 %, thoracoscopy: 1.0 %; *P* = 1.00). In the open group, one patient died 29 days postoperatively because of respiratory failure due to aspiration pneumonia. In the thoracoscopy group, one patient was reoperated 7 days postoperatively because of gastric conduit ischemia; however, he died 9 days after the initial surgery because of septic shock.

### Reoperation

As shown in Table 4, reoperations were performed more frequently in the thoracoscopy group (open: 1.8 %, thoracoscopy: 9.9 %; *P* = 0.016). Patients who underwent laparoscopic surgery also underwent reoperations more frequently than those who underwent open abdominal surgery; however, the difference was insignificant (open: 4.1 %, laparoscopy: 9.2 %; *P* = 0.20). These reoperations were performed in six patients who underwent a combination of thoracoscopic and laparoscopic esophagectomy: four patients who underwent a combination of thoracoscopic and open abdominal esophagectomy and two patients who underwent a combination of open chest and open abdominal esophagectomy. Associations between these reoperations and baseline characteristics are shown in Table 3. Our analysis demonstrated that the only risk factor for reoperations was the thoracoscopic approach.

**Table 4:** Complications causing reoperation

Abdominal approach	Thoracic approach	
	Open (*n* = 2)	Thoracoscopy (*n* = 10)
Open (*n* = 6)	Gastric conduit ischemia	Mediastinal abscess not related to leak
	Thoracic bleeding	Chylous leak from thoracic duct
		Mechanical obstruction of jejunum
		Pancreatic juice leak
Laparoscopy (*n* = 6)		Air leak from a bulla on the right lung
		Cervical abscess related to leak
		Gastric conduit ischemia
		Transhiatal herniation of colon
		Acute cholecystitis
		Omental necrosis

### Laboratory abnormalities

There was no difference in maximum median white blood cell count after esophagectomy (open: 12,200/mL, thoracoscopy: 11,920/mL; *P* = 0.63). Grade 3 and 4 laboratory abnormalities after esophagectomy are shown in Table 5. Among these abnormalities, elevated alanine aminotransferase was observed more often in the open group; however, the difference was not statistically significant (open: 25.7 %, laparoscopy: 16.8 %; *P* = 0.13).

**Table 5:** Laboratory abnormalities after esophagectomy

Grade 3 or 4 Abnormalities	Open (*n* = 109)		Thoracoscopy (*n* = 101)		*P* ^a^
	*n*	%	*n*	%	
Decreased white blood cell count	0	0	0	0	N/A
Decreased hemoglobin	11	10.1	5	5.0	0.20
Decreased platelet count	1	0.9	0	0	1.00
Increased blood bilirubin	9	8.3	10	9.9	0.81
Increased AST	10	9.2	10	9.9	1.00
Increased ALT	28	25.7	17	16.8	0.13
Increased Creatinine	2	1.8	0	0	0.50

*N/A* not applicable, *AST* aspartate aminotransferase, *ALT* alanine aminotransferase

^a^Fisher’s exact test

## Discussion

This prospective multicenter study demonstrated that the incidences of intraoperative complication, overall postoperative complication, and mortality did not differ between the two approaches for esophagectomy. To the best of our knowledge, this is the third prospective multicenter study that evaluated the safety profile of thoracoscopic esophagectomy. The first phase II multicenter trial was conducted by Luketich et al. [10]. They reported that minimally invasive esophagectomy (MIE, a combination of thoracoscopic and laparoscopic esophagectomy) performed in 99 patients with esophagogastric adenocarcinoma resulted in a 30-day mortality rate of 2 %, with the incidences of 4.9 and 7.8 % for pneumonia and anastomotic leak, respectively. Subsequently, the first phase III multicenter randomized trial was conducted by Biere et al. [9], who compared 59 cases of MIE with 56 cases of open esophagectomy. In this trial, the incidence of pulmonary infection within 14 days postoperatively (the primary endpoint) was significantly lower in MIE (9 %) than in open esophagectomy (29 %). The trial also showed no significant difference in mortality between MIE (3 %) and open esophagectomy (2 %).

In the present study, the frequency of reoperation was higher in thoracoscopy (9.9 %) than in open esophagectomy (1.8 %). Similarly, a Japanese web-based nationwide study that included more than 5,000 patients reported that thoracoscopic and/or laparoscopic esophagectomy was associated with a higher frequency of reoperation (8.0 %) than open esophagectomy (5.6 %) [8]. Limited access and insufficient traction in thoracoscopic surgery could cause unexpected complications that require reoperation. In contrast, the first prospective phase III trial reported no significant difference in the frequency of reoperation between the open (11 %) and MIE groups (14 %), where only surgeons who had performed 10 or more MIEs were responsible for both modalities [9]. The enrollment period for the JCOG0502 trial extended from December 2006 to February 2013. Most participating institutes introduced thoracoscopic esophagectomy as a new technique during this study period. As thoracoscopic esophagectomy requires greater expertise and a long learning curve before getting stable results [17, 18], the higher frequency of reoperation may be attributable to the low level of experience with a new technique during the learning period. Our ongoing phase III study (JCOG1109, started in November 2012), which compares three preoperative therapies for locally advanced esophageal cancer [19], permits surgeons to use the thoracoscopic approach, similar to the JCOG0502 trial. However, in contrast to JCOG0502, only surgeons credentialed by the study chair are permitted to perform thoracoscopic surgery. Each credentialed surgeon should have received certification (or its equivalent) from the Japan Society for endoscopic surgery and should have performed 30 or more thoracoscopic esophagectomies. We expect these stringent criteria will significantly decrease the frequency of reoperation in the JCOG1109 trial.

The British population-based national study that included more than 7,000 patients reported the same trend with regard to reoperation: Thoracoscopic and laparoscopic esophagectomy were both associated with a higher frequency of reoperation (8.8 %) than open esophagectomy (5.6 %) [20]. Further analysis showed that the frequency of reoperation after the combination of thoracoscopic and laparoscopic esophagectomy was 10.4 % compared with 8.3 % after thoracoscopic or laparoscopic esophagectomy alone. Similarly, in the present study, the frequency of reoperation after the combination of thoracoscopic and laparoscopic esophagectomy was 10.3 versus 8.0 % after either thoracoscopic or laparoscopic esophagectomy. Moreover, four out of six reoperations after laparoscopic surgery were performed because of damage to the abdominal organs (Table 4). Although the laparoscopic approach was not classified as a significant risk factor for reoperation (Table 3), thoracoscopic esophagectomy in combination with the laparoscopic approach appears to increase the risk of reoperation.

In the present study, atelectasis and pneumonia were less common in the group that underwent thoracoscopic esophagectomy. It is well known that thoracoscopic esophagectomy decreases pulmonary complications compared with open esophagectomy [3, 5, 9, 20]. However, whether the prone position during thoracoscopic surgery is responsible for this decrease remains controversial [21]. The prone position with artificial pneumothorax is reported to have the advantage of avoiding total lung collapse over the lateral decubitus position, thereby decreasing pulmonary complications [22, 23]. However, the prone position failed to demonstrate any superiority in the prevention of atelectasis and/or pneumonia in the present study. On the basis of this observation, we believe that atelectasis occurred less often in the thoracoscopy group, not because of body position but because this surgical approach decreased the extent of chest trauma. Consequently, postoperative pain and discomfort were minimized, allowing patients to take deep breaths. However, as majority of patients (98 %) in the thoracoscopy group underwent tracheal intubation with one-lung ventilation, it remains unclear whether a combination of the prone position with artificial pneumothorax and single-lumen tracheal intubation would decrease pulmonary complications.

When compared to the previously reported prospective multicenter studies, the advantage of the present study is its homogeneous patient population. All patients were diagnosed with clinical stage T1bN0M0 thoracic esophageal cancer, and all underwent esophagectomy without preoperative chemotherapy and/or radiotherapy. Thus, we could precisely evaluate the safety of the thoracoscopic approach without any staging or treatment interactions. Nevertheless, this study reflects an inherent limitation: because it was designed as a nonrandomized comparison, results could be affected by patient selection bias and combination bias in the thoracoscopy group toward the laparoscopic surgery. This study also may reflect the low level of experience during the learning period for thoracoscopic esophagectomy. Therefore, we are now planning a multicenter randomized phase III trial (JCOG1409) to confirm the efficacy and safety of thoracoscopic esophagectomy performed by the credentialed surgeons.

## Conclusions

In conclusion, the present study demonstrated that thoracoscopic esophagectomy was a safe procedure with morbidity and mortality comparable with those of open esophagectomy. However, thoracoscopic esophagectomy was associated with a higher frequency of reoperation. Therefore, surgeons with little experience should take extra precautions to avoid any postoperative complications that may require reoperation.
